# Comparison of intervertebral disc and nucleus pulposus stresses under different lumbar curvature configurations

**DOI:** 10.3389/fbioe.2026.1969400

**Published:** 2026-10-23

**Authors:** Shengbo Niu, Huan Yang, Jianghong Wu, Lin Song, Bingkun Meng, Zehao Niu, Qudi Zhang, Xuhui Zhang

**Affiliations:** 1 Department of Orthopedics, The 83rd Group Army Hospital, Xinxiang, Henan, China; 2 Department of Spine Surgery, The First Affiliated Hospital of Naval Medical University, Shanghai, China; 3 Department of Emergency Medicine, The Second Affiliated Hospital of Army Medical University, Chongqing, China; 4 Department of Orthopedics, Liaocheng Infectious Diseases Hospital, Liaocheng, Shan Dong, China; 5 Department of Orthopedics, The First Affiliated Hospital of Naval Medical University, Shanghai, China; 6 Department of Burns and Plastic Surgery, The 83rd Group Army Hospital, Xinxiang, Henan, China

**Keywords:** finite element analysis, intervertebral disc, lumbar curvature configuration, nucleus pulposus, von Mises stress

## Abstract

**Background:**

To compare the intervertebral disc and nucleus pulposus stresses under different lumbar curvature configurations.

**Methods:**

Finite element models of a healthy lumbar spine were established for eight postures: standing with a forward inclination position, standing in a neutral position, standing with a backward inclination position, relaxed sitting with a forward inclination position, upright sitting in a neutral position, relaxed sitting with a backward inclination position, relaxed sitting in a neutral position, and relaxed sitting in a neutral position with lumbar support. After assigning different material properties, the model of the standing in neutral position was selected for validation. Following the application of a 400 N physiological load, the lumbar intervertebral disc and nucleus pulposus von Mises stresses were analyzed for each posture.

**Results:**

Among the three standing postures, the peak von Mises stresses in the L3-L4, L4-L5, and L5-S1 discs were higher than that in the L1-L2 and L2-L3 discs. The smallest peak von Mises stresses in the L3-L4, L4-L5, and L5-S1 discs were observed in the neutral standing position. The nucleus pulposus stresses were minimal in the L3-L4 and L4-L5 discs in the neutral standing position, while the nucleus pulposus stresses in L5-S1 disc was minimal in the backward-inclined standing position. Among the five sitting postures, the smallest peak von Mises stress and nucleus pulposus stresses in the L4-L5 and L5-S1 discs were found in the upright sitting in a neutral position and the relaxed sitting in a neutral position with lumbar support. Across all postures, the nucleus pulposus stresses was the lowest.

**Conclusion:**

Lumbar curvature configurations significantly influences both the distribution and magnitude of von Mises stress in the L4-L5 and L5-S1 intervertebral discs and nucleus pulposus. Maintaining proper spinal alignment and avoiding postures that reduce lumbar lordosis are generally more favorable for lowering stress levels at these two segments.

## 1 Introduction

Mechanical loading is a significant factor in the etiology of low back pain. The configurations of spinal structures and the sagittal curvature of the lumbar spine determine its mechanical behavior. Understanding the impact of changes in lumbar sagittal alignment on lumbar intervertebral disc loading is crucial for the assessment of low back pain (Stott and Driscoll, 2023). Targeted postural adjustments can influence conservative treatment and surgical planning for patients with spinal disorders (Fasser et al., 2025). The L4-L5 and L5-S1 levels are most clinically relevant to the development and progression of spinal problems (Lund et al., 2026). Elucidating the effects of lumbar curvature configurations in different postures on lumbar disc loading at L4-L5 and L5-S1 is of great importance. The magnitude and distribution of lumbar intervertebral disc loads depend on various factors, including but not limited to gender, body weight, posture, and anatomical structure. For example, in unsupported sitting, intradiscal pressure (IDP) increases by approximately 30% compared with upright standing (Roman-Liu et al., 2023). Changes in IDP during both standing and sitting are closely correlated with trunk flexion angle, and the increase in IDP at the same flexion angle is more pronounced in the standing position (Roman-Liu et al., 2023). While implanting pressure sensors into human lumbar discs to measure IDP under different postures can yield more accurate values, this invasive method is not widely applicable and cannot comprehensively represent the distribution of disc loading. Biomechanical testing using cadaveric spine specimens provides high-level evidence (Crawford et al., 2025), however, obtaining such specimens is difficult, and it is challenging to replicate the true *in vivo* curvature. Furthermore, biomechanical testing of spinal specimens must be conducted under load or displacement control, making it difficult to apply repeated or varying loading conditions to the same specimen. Finite element analysis (FEA) can circumvent the limitations of these two methods. FEA utilizes finite element models (FEMs) to simulate lumbar spine anatomy by assigning material parameters to different structures, enabling the calculation of load magnitudes at any location within the lumbar spine and obtaining all mechanical field variables for any given value. Von Mises stress is a scalar metric derived from principal stresses, used to simplify multiaxial stress states for comparison and to output stress distributions. The von Mises stress in the intervertebral disc and nucleus pulposus is primarily obtained through FEA (Ardatov et al., 2026). A study utilized Anybody Modeling System to develop a musculoskeletal model of the cervical spine to investigate the effects of neutral posture on mechanical properties of cervical spine under different gravitational conditions, and the results revealed that under microgravity, both the compressive and shear forces acting on the cervical intervertebral discs were reduced during neutral standing posture, while the shear force direction in the C6C7 and C7T1 segments changed (He et al., 2025).

Changes in sagittal profile affect spinal load distribution and range of motion. Changes in lumbar lordosis (LL) significantly alter the loading patterns of the intervertebral disc and facet joints; in healthy populations, a greater LL is associated with a decreasing trend in disc compressive force, whereas facet joint loading tends to increase (Müller et al., 2021). Appropriate lumbar support can increase the LL and reduce lumbar back muscle activity and associated spinal loading (Gao et al., 2023). Incorporating patient-specific sagittal profiles into FEMs may provide valuable insights for biomechanical analysis and targeted treatment (Stott and Driscoll, 2023). To our knowledge, limited studies have employed FEA to analyze lumbar disc von Mises stresses under different standing and sitting lumbar curvature configurations, largely due to the constraint that most computed tomography (CT) or magnetic resonance imaging (MRI) scans are performed in the supine position. Radiographic findings under axial loading in the upright position differ significantly from those in the unloaded supine position (Sehic et al., 2022). Based on the conventional method of constructing FEMs from CT data, this study referenced the actual lumbar curvature configurations presented in lateral lumbar X-rays of different standing and sitting postures to establish lumbosacral bone-ligament FEMs. By analyzing lumbar disc von Mises stresses during commonly adopted standing and sitting postures in daily life to provide a reference for adopting correct postures in individuals without low back pain symptoms or other spinal disorders.

## 2 Materials and methods

### 2.1 Study subject

A 44-year-old male adult volunteer (height: 168 cm, weight: 76 kg, body mass index: 26.9) without symptoms of low back pain or other spinal disorders was recruited. The following conditions were considered exclusion criteria: (1) radiographic evidence of spinal deformity, rotation, or slippage of lumbar vertebral bodies; (2) presence of tumors, infections, osteoporosis, neuromuscular diseases, or systemic diseases (e.g., rheumatism); (3) mental or psychological disorders or pregnancy precluding radiographic examination; and (4) abnormalities of the hip, knee, or ankle joints or leg length discrepancy. Written informed consent was obtained, and this research has been approved by the authors’ affiliated institutions.

### 2.2 Data collection

CT scanning of the volunteer’s lumbosacral spine in the supine position was performed to obtain data in Digital Imaging and Communications in Medicine (DICOM) format. To minimize radiation exposure, lateral X-ray images in different postures were acquired in two separate sessions at 2-week intervals, 1 month after the CT scan. Standing postures included: standing with the trunk flexed forward approximately 30° (Figure 1A), standing in a neutral position (Figure 1B), and standing with the trunk extended backward approximately 15° (Figure 1C). Sitting postures included: sitting with the lumbar spine relaxed and the trunk flexed forward approximately 30° (Figure 1D), sitting upright in a neutral position (Figure 1E), sitting with the lumbar spine relaxed and the trunk extended backward approximately 15° (Figure 1F), sitting with the lumbar spine relaxed in a neutral position (Figure 1G), and sitting with the lumbar spine relaxed in a neutral position with lumbar support (a rigid support backrest measuring 40 cm in length, 35 cm in width, and 10 cm in height) (Figure 1H).

**FIGURE 1:**
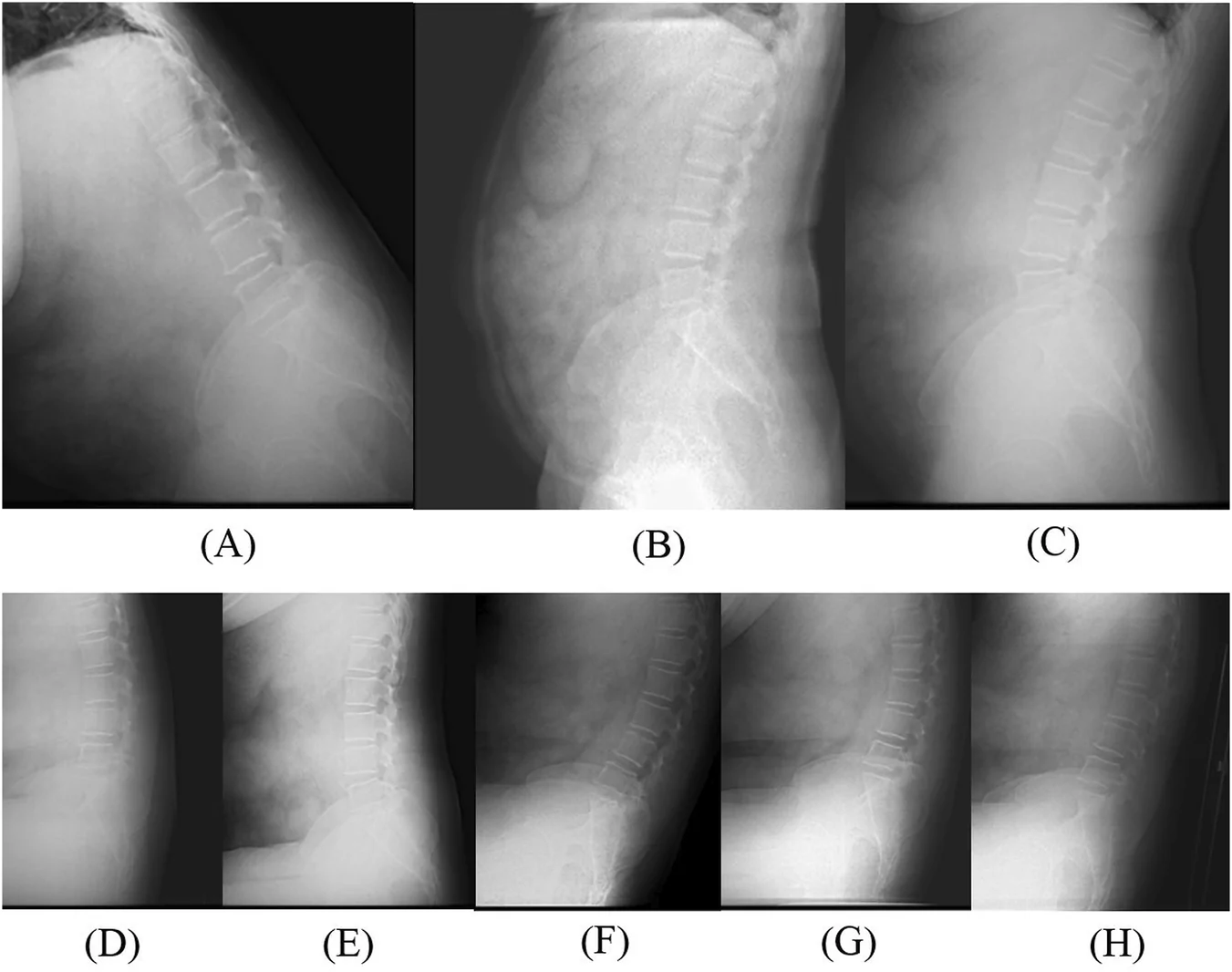
X-ray films of three standing and five sitting lumbar curvature configurations. **(A)** Standing with a forward inclination position, **(B)** standing in a neutral position, **(C)** standing with a backward inclination position, **(D)** relaxed sitting with a forward inclination position, **(E)** upright sitting in a neutral position, **(F)** relaxed sitting with a backward inclination position, **(G)** relaxed sitting in a neutral position, **(H)** relaxed sitting in a neutral position with lumbar support.

### 2.3 Reconstruction of lumbosacral spine solid models under different standing and sitting lumbar curvature configurations

The supine lumbosacral spine DICOM data were imported into the finite element modeling software Mimics. Based on differences in grayscale values representing various tissue components, threshold segmentation and region growing were performed to isolate the lumbosacral region. Operations such as wrap and smoothing were then applied to fill surface holes and smooth the model surface, restoring the original morphology of the lumbar spine and establishing an STL triangular mesh model of the lumbosacral vertebral segments. The generated STL model was imported into the reverse engineering software Geomagic Studio for polygon mesh repair. The model surface was relaxed and smoothed, and self-intersecting surfaces and small holes were removed. By adjusting the U-V grid lines of each closed contour (patch), the surface curvature of the model was regulated, ultimately generating a Non-Uniform Rational B-Splines (NURBS) surface solid model with GI continuity.

Conventional modeling methods are nearly incapable of obtaining valid data for standing or sitting postures for modeling due to the limitations of CT acquisition positioning. The reconstruction and modeling approach in this study was as follows: a lumbosacral spine solid model was first established using supine CT data. Then, referencing the positions and angles of the vertebral bodies in lateral X-ray images of different postures, each vertebral body in the lumbosacral model was treated as a “component unit.” The sagittal angles of the vertebral bodies and the heights of the intervertebral spaces were adjusted to rearrange the spatial positions of the lumbosacral vertebral bodies in the sagittal plane, thereby rapidly constructing three-dimensional finite element models that could represent the true lumbar curvature in different postures. The specific procedure was as follows: (1) The IGS models of lumbar vertebrae L1-S1 were imported into SolidWorks, converted into “component units,” and saved in SLDPRT format. The saved components were then imported into a SolidWorks assembly and aligned (Figures 2A,B). (2) Lateral lumbar X-ray images of different standing and sitting postures were imported into CAD software. A coordinate system was established with the anterosuperior corner of S1 as the origin. The angles between the lines connecting the anterosuperior and anteroinferior corners of the L1-L5 vertebral bodies and the vertical axis of the coordinate system were measured. Simultaneously, the heights of the L1-S1 intervertebral spaces were measured (Figures 2C–F). (3) The assembly was readjusted based on the measured vertebral body angles and intervertebral space heights. Finally, the entire assembly was rotated according to the angle between the superior endplate of the S1 vertebral body and the horizontal axis of the coordinate system. This resulted in the establishment of IGS models whose curvatures matched those observed in the lateral X-ray images of the different postures Figures 2G,H).

**FIGURE 2:**
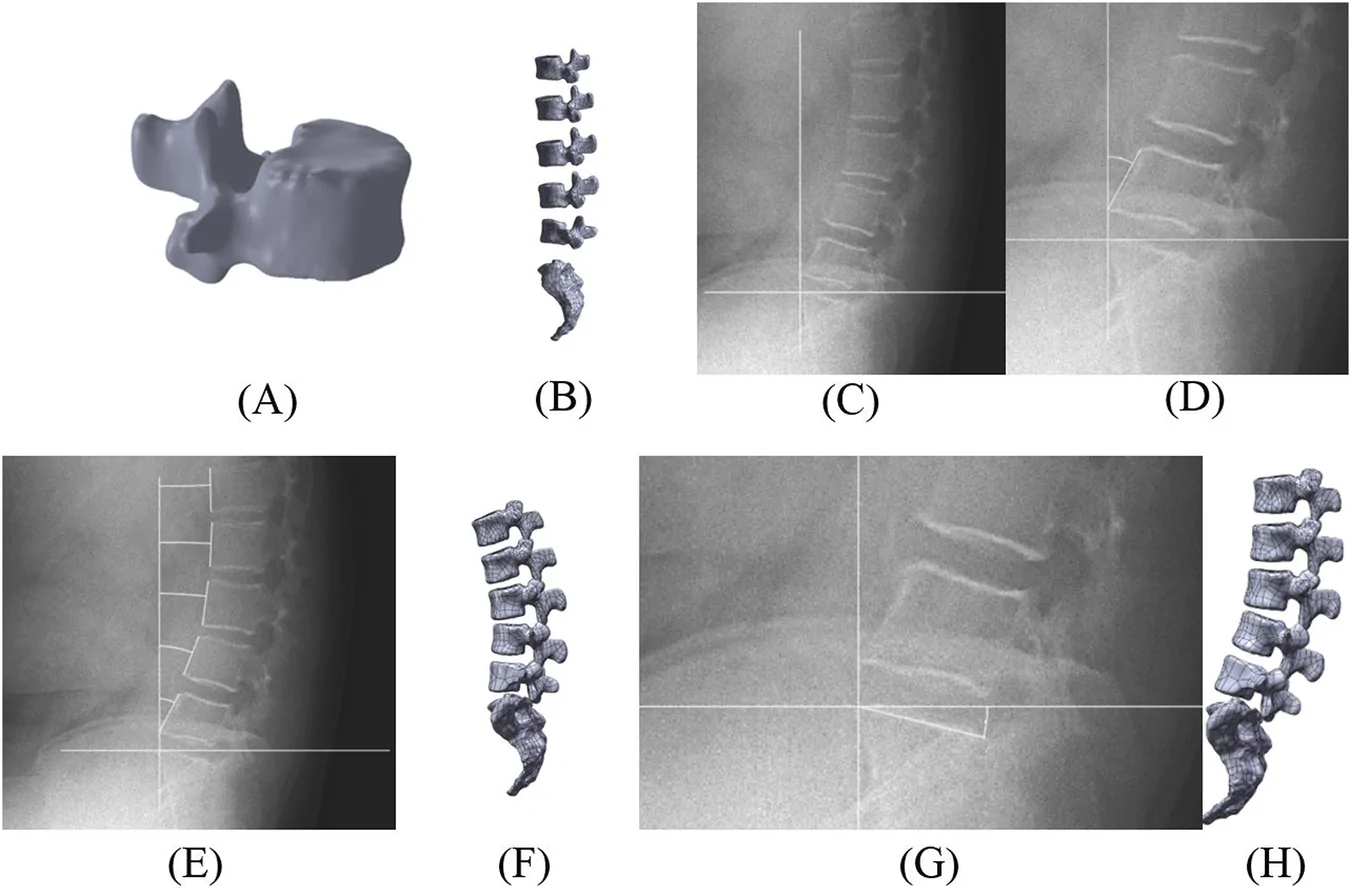
Establishment of igs models of lumbar vertebrae under different lumbar curvature configurations **(A–H)**.

### 2.4 Establishment of lumbosacral spine finite element models under different standing and sitting lumbar curvature configurations

The modified IGS models from SolidWorks were imported into HyperMesh. The vertebral bodies were meshed with tetrahedral elements (C3D4). The outermost 1 mm of the mesh was defined as cortical bone, with the remaining portion defined as cancellous bone. Based on the inferior and superior surfaces of the vertebral bodies and using contour lines as mapping paths, the intervertebral disc tissue was generated. The uppermost and lowermost elements of the created intervertebral disc were defined as the endplates. The remaining portion of the disc, after removing the endplates, was defined from the outer layers inward (1–8 layers of elements) as the annulus fibrosus, with the central portion defined as the nucleus pulposus (Eberlein et al., 2001). To investigate the von Mises stress distribution characteristics of the nucleus pulposus and its variation trends under different lumbar curvature configurations, this study performed von Mises stress analysis in a complete intervertebral disc finite element model, after which the nucleus pulposus was isolated as a sub-region from the overall disc model and its von Mises stress was output separately. As the vertebral bodies and intervertebral discs had different mesh types and sizes, they were structurally unrelated after creation. Following the establishment of the intervertebral discs, Tie contact constraints were created to connect the vertebral body and intervertebral disc meshes. Spinal ligaments, including the anterior longitudinal ligament, posterior longitudinal ligament, interspinous ligament, supraspinous ligament, ligamentum flavum, intertransverse ligament, and capsular ligament, were simulated using spring elements. All ligaments were assumed to be isotropic, homogeneous, continuous, and linearly elastic materials. Relevant parameters such as elastic modulus and Poisson’s ratio were set in the software. Specific parameters can be referenced in Table 1. Finite element models of a healthy lumbar spine were established for eight postures (Supplementary Figures S1, 2). The intervertebral discs were meshed with a global element size of 1 mm, which was verified by a mesh convergence analysis.

**TABLE 1:** Material parameters of the main structures of the lumbar spine.

Material	E (MPa)	PR	CSR (mm^2)^	References
Cortical bone	12000	0.3	—	Nie et al. (2009) Ruberté et al. (2009) Goel et al. (1988) Sharma et al. (1995)
Cancellous bone	100	0.2	—	Nie et al. (2009) Goel et al. (1988)
Posterior bony structures	3,500	0.25	—	Shirazi-Adl et al. (1986)
Cartilaginous endplate	23.8	0.4	—	Schmidt et al. (2007) Ueno and Liu (1987) Lu et al. (1996a)
Annulus fibrosus	450	0.3	—	Nie et al. (2009)
Annulus fibrosus matrix	4.2	0.45	—	Clin et al. (2019) Nie et al. (2009) Schmidt et al. (2007)
Nucleus pulposus	0.2	0.49	—	Sharma et al. (1995) Schmidt et al. (2007)
ALL	20	0.3	63.7	Lu et al. (1996b) Nie et al. (2009) Goel et al. (1988) Polikeit et al. (2003)
PLL	20	0.3	20	
FL	19.5	0.3	40	
SL	15	0.3	30	
ISL	11.6	0.3	40	
ITL	58.7	0.3	1.8	
CL	32.9	0.3	30	

E: Young’s modulus; PR: Poisson’s ratio; CSR: Cross sectional area; ALL: Anterior longitudinal ligament; PLL: Posterior longitudinal ligament; FL: flavum ligament; SL: supraspinous ligament; ISL: Interspinous ligament; ITL: Intertransverse ligament; CL: capsular ligament.

### 2.5 Validation of the finite element model

This study validated the biomechanical performance using the established neutral standing posture model as a representative. A moment of 7.5 N·m was applied to primarily test the primary motion range of motion of the model at the L2-L3, L3-L4, and L4-L5 levels in the sagittal, coronal, and transverse planes. The results demonstrated that the finite element model developed in this study yielded outcomes close to those of previous studies, effectively simulating the biomechanical behavior of the lumbar spine under load. Although certain numerical differences were observed, these may be attributed to individual variability, differences in model parameter settings, and numerical processing methods. Thus, the model was deemed suitable for subsequent analyses (Table 2; Figure 3).

**TABLE 2:** Comparison of ROM at the L2-L3, L3-L4, and L4-L5 between this study and previous studies.

PB	L2-L3 ROM (°)				L3-L4 ROM (°)				L4-L5 ROM (°)			
	This study	Li et al. (2009)	Renner et al. (2007)	Panjabi et al. (1994)	This study	Li et al. (2009)	Renner et al. (2007)	Panjabi et al. (1994)	This study	Li et al. (2009)	Renner et al. (2007)	Panjabi et al. (1994)
FE	7.242	5.4	6.54	8.6	8.384	4.3	9.9	8.4	9.622	1.9	13.75	11.5
LB	3.539	2.9	12	9.5	4.020	3.4	9.75	10	3.731	4.7	10.35	8.3
AR	1.634	2.5	1.10	2.8	2.789	2.4	1.61	3.2	2.049	2.9	3.9	1.8

ROM: Range of motion; PB: primary motion; FE: flexion extension; LB: lateral bending; AR: axial rotation.

**FIGURE 3:**
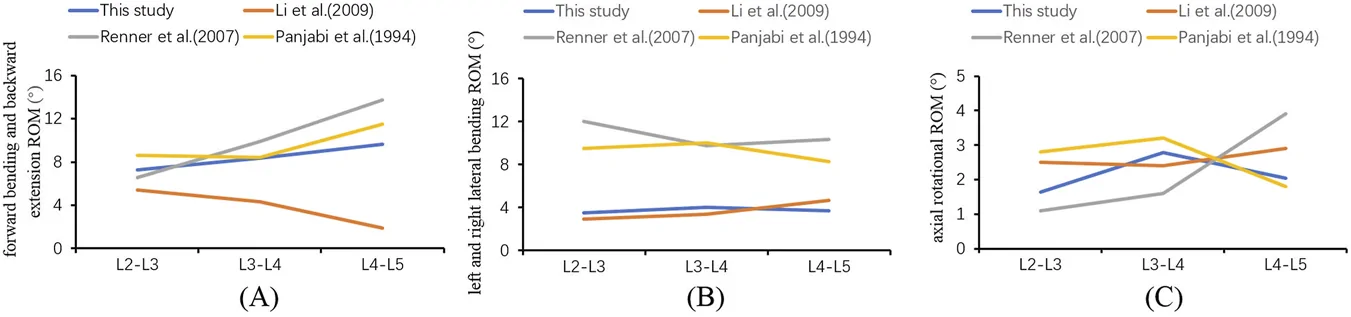
Verification of the model’s ROM in forward flexion and backward extension **(A)**, left and right lateral flexion **(B)**, and rotation **(C)**. ROM: range of motion.

### 2.6 Mesh convergence analysis

To verify that the predicted disc stresses were independent of mesh density, a mesh convergence analysis was performed on the intervertebral disc models of the L4-L5 and L5-S1 segments, the two levels of primary clinical relevance. For each disc, three meshes of increasing density were generated in HyperMesh by setting the global element size to 2 mm, 1 mm, and 0.5 mm, with proportional refinement of the endplates, annulus fibrosus, and nucleus pulposus. Under the 400 N compressive physiological load, the peak von Mises stress of the L4-L5 and L5-S1 discs was compared across the three meshes. Refining the mesh from 2 mm to 1 mm decreased the peak von Mises stress of the L4-L5 disc from 2.932 MPa to 2.754 MPa and that of the L5-S1 disc from 2.978 MPa to 2.825 MPa, whereas further refinement from 1 mm to 0.5 mm changed these values by only 0.04% and 0.07%, respectively. The predicted disc stresses can therefore be considered independent of mesh density at the 1 mm discretization, and the 1 mm element size was adopted for the intervertebral discs in all postural models.

During the loading process of the lumbar spine in various standing and sitting postures, the volunteer’s sacrum served as a relatively fixed reference position. Therefore, in the finite element model, translations and rotations on both sides of the sacrum were constrained to ensure stable support during model loading. Simultaneously, a coupling constraint reference point was established on the superior surface of the L1 vertebral body. A vertical load of 400 N was applied at this point as the loading force, which is equivalent to the physiological load of the upper body weight (Patwardhan et al., 2021; Guo et al., 2025). However, spinal loading in real life is often complex, involving six-axis loading, and a constant load alone cannot fully replicate the true mechanical environment of daily activities (Holsgrove and Ebisch, 2025). In this study, a standardized boundary condition and loading protocol was adopted primarily to enable controlled comparisons across different postures, thereby allowing a clearer identification of the effects of posture itself on intervertebral disc and nucleus pulposus stresses.

## 3 Results

### 3.1 Distribution and magnitude of intervertebral disc and nucleus pulposus stresses under different standing lumbar curvature configurations

As the torso leaned forward from a standing posture, the distribution of von Mises stress in the lumbar intervertebral discs and nuclei pulposi shifts from posterior to anterior, remaining largely symmetrical left and right (Figure 4; Supplementary Figure S3). Among the three standing postures, the peak von Mises stresses in the L3-L4, L4-L5, and L5-S1 discs were higher than those in the L1-L2 and L2-L3 discs. The smallest peak von Mises stresses in the L3-L4, L4-L5, and L5-S1 discs were observed in the neutral standing position. The smallest nucleus pulposus stresses in the L3-L4 and L4-L5 discs were found in the neutral standing position, while the smallest nucleus pulposus stress in the L5-S1 disc was observed in the backward-inclined standing position (Table 3; Table 4; Figures 5, 6). Across all standing postures, the nucleus pulposus stresses were the lowest.

**FIGURE 4:**
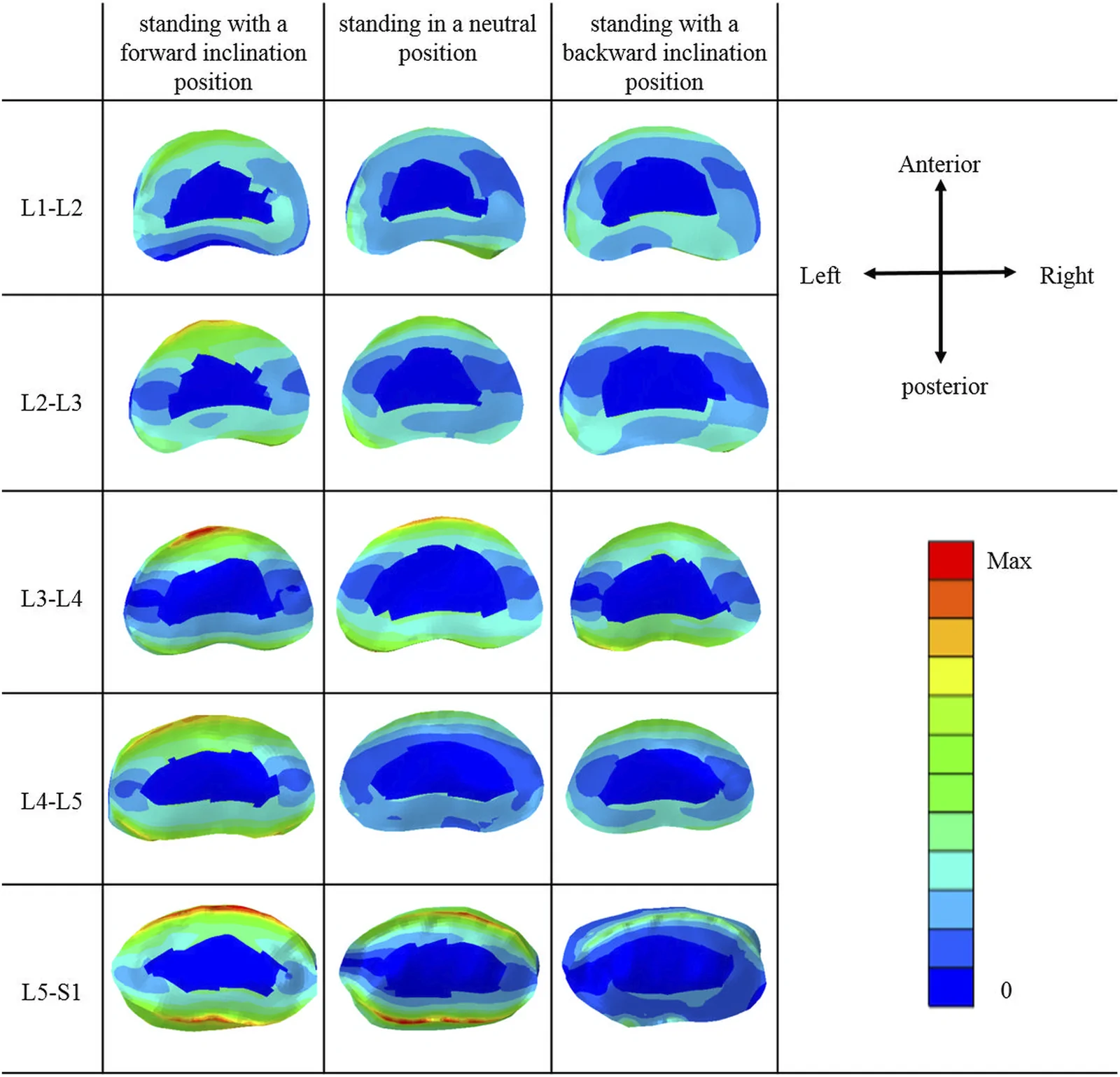
The Mises stress distribution of the lumbar intervertebral discs under different standing lumbar curvature configurations. MAX: maximum.

**TABLE 3:** Peak Mises stresses of lumbar intervertebral discs under different standing and sitting lumbar curvature configurations (MPa).

Lumbar curvature configurations	L1-L2	L2-L3	L3-L4	L4-L5	L5-S1
Standing with a forward inclination position	1.026	2.038	3.275	2.852	3.177
Standing in a neutral position	1.157	1.434	2.847	2.754	2.825
Standing with a backward inclination position	1.220	1.687	2.957	2.947	3.106
Relaxed sitting with a forward inclination position	0.486	0.985	1.036	1.927	2.202
Upright sitting in a neutral position	0.459	0.472	0.735	0.623	0.669
Relaxed sitting with a backward inclination position	0.370	0.557	1.058	1.416	1.875
Relaxed sitting in a neutral position	0.277	0.303	0.371	0.762	1.166
Relaxed sitting in a neutral position with lumbar support	0.372	0.727	0.763	0.674	0.684

**TABLE 4:** Peak Mises stresses of nuclei pulposi under different standing and sitting lumbar curvature configurations (MPa).

Lumbar curvature configurations	L1-L2	L2-L3	L3-L4	L4-L5	L5-S1
Standing with a forward inclination position	0.074	0.115	0.100	0.155	0.119
Standing in a neutral position	0.061	0.065	0.086	0.088	0.114
Standing with a backward inclination position	0.055	0.084	0.104	0.179	0.102
Relaxed sitting with a forward inclination position	0.023	0.028	0.053	0.087	0.091
Upright sitting in a neutral position	0.020	0.021	0.021	0.022	0.022
Relaxed sitting with a backward inclination position	0.018	0.032	0.043	0.066	0.060
Relaxed sitting in a neutral position	0.009	0.011	0.019	0.036	0.058
Relaxed sitting in a neutral position with lumbar support	0.014	0.018	0.021	0.020	0.011

**FIGURE 5:**
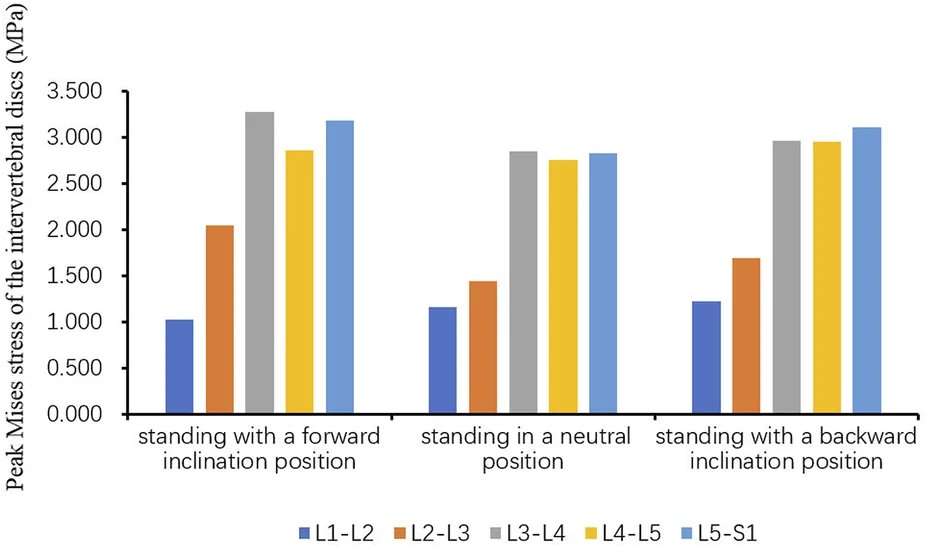
The peak Mises stresses of the lumbar intervertebral discs under different standing lumbar curvature configurations.

**FIGURE 6:**
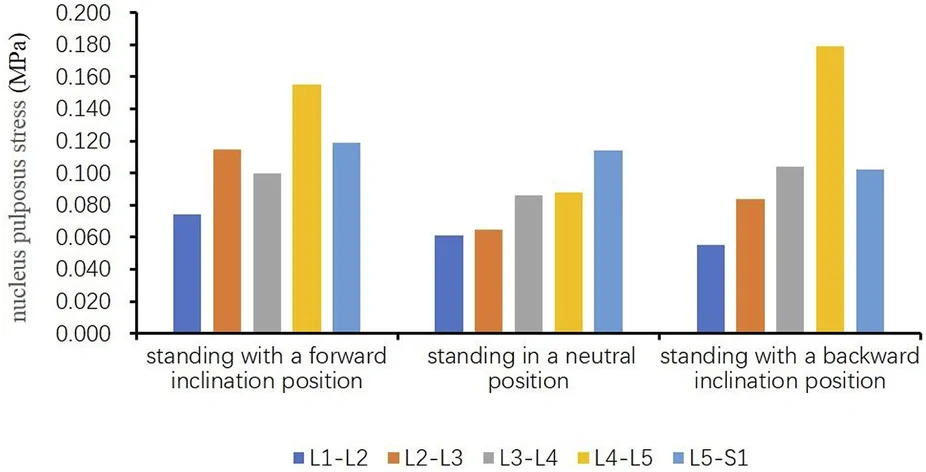
The peak Mises stresses of the nuclei pulposi under different standing lumbar curvature configurations.

### 3.2 Distribution and magnitude of intervertebral disc and nucleus pulposus stresses under different sitting lumbar curvature configurations

Compared to the relaxed sitting with a forward inclination and a backward inclination position, the distribution of von Mises stress in the lumbar intervertebral discs and nuclei pulposi in the upright sitting in a neutral position was primarily concentrated in the posterior region. In contrast, the stress distribution in the relaxed sitting in a neutral position was located more anteriorly than in the upright sitting in a neutral position. When lumbar support was provided, the stress distribution was mainly concentrated in the posterior region and maintained an approximately bilateral symmetry (Figure 7; Supplementary Figure S4). The smallest peak von Mises stresses and nucleus pulposus stresses in the L4-L5 and L5-S1 lumbar discs were observed in both the upright sitting in a neutral position and the relaxed sitting in a neutral position with lumbar support (Table 3; Table 4; Figures 8, 9). Across all sitting postures, the nucleus pulposus stresses were the lowest.

**FIGURE 7:**
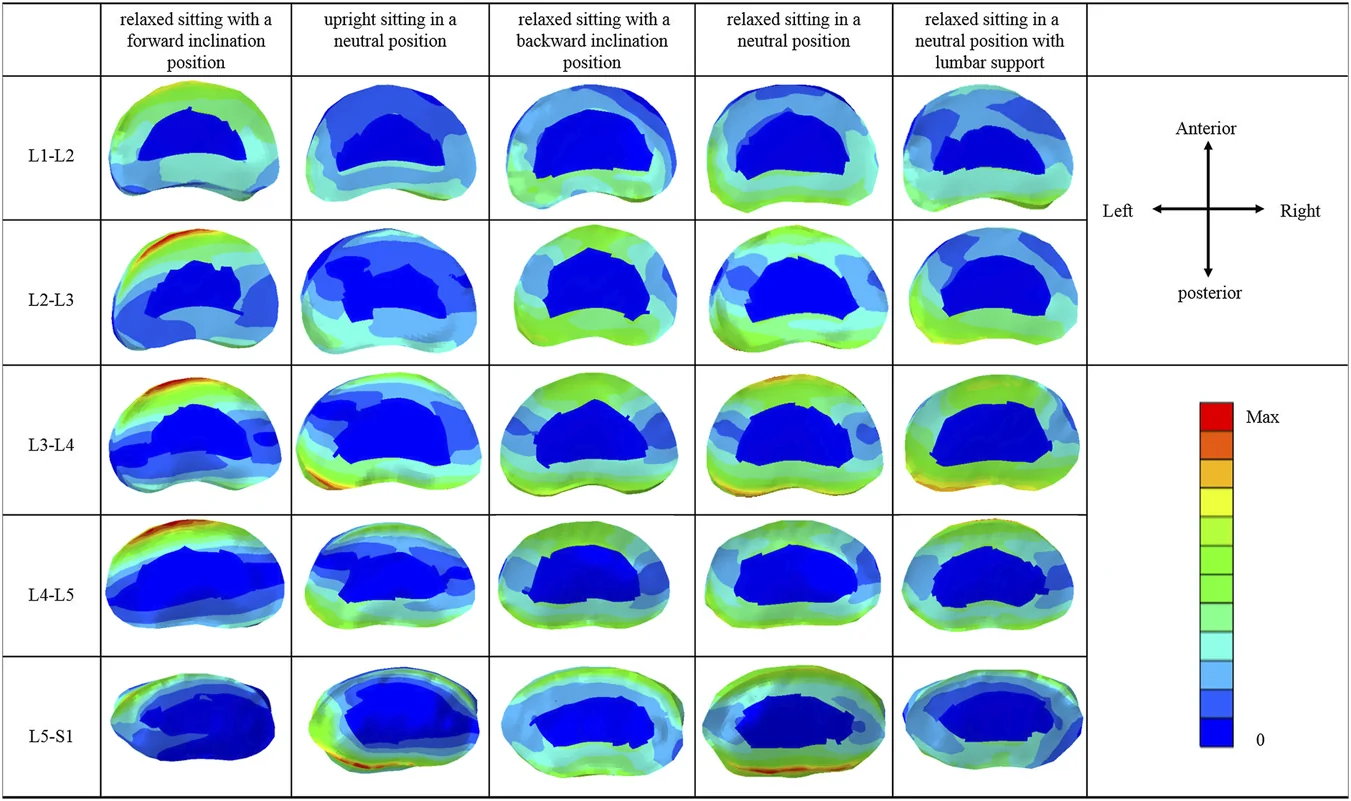
The Mises stress distribution of lumbar intervertebral discs under different sitting lumbar curvature configurations. MAX: maximum.

**FIGURE 8:**
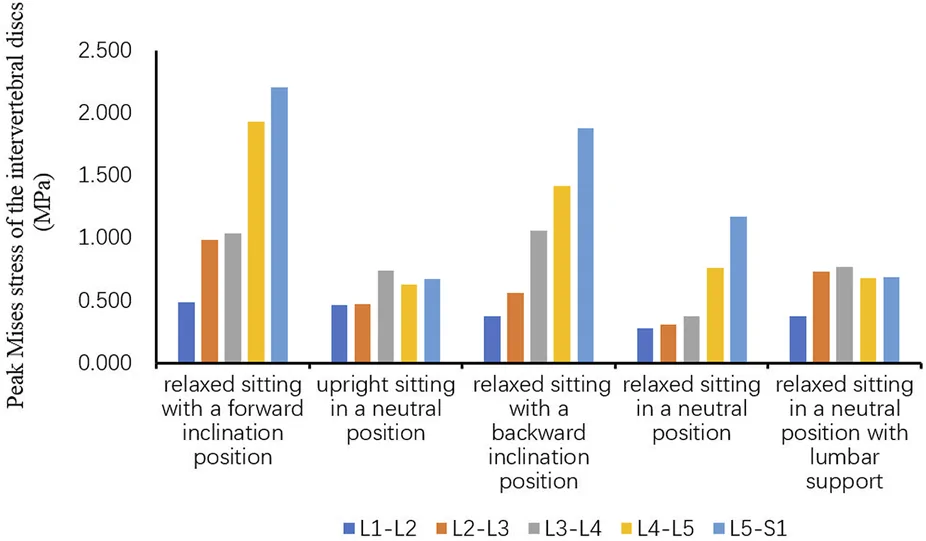
The peak Mises stresses of the lumbar intervertebral discs under different sitting lumbar curvature configurations.

**FIGURE 9:**
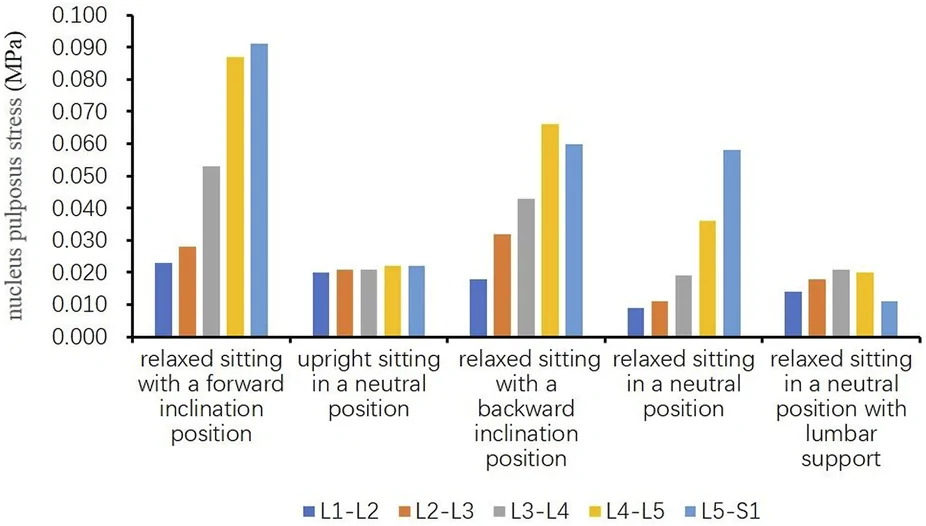
The peak Mises stresses of the nuclei pulposi under different sitting lumbar curvature configurations.

## 4 Discussion

The computational parameters of the FEMs primarily represent trends, and their absolute values may not be accurate. However, the stress distribution patterns they reflect can effectively explain the biomechanical mechanisms underlying disease onset and progression (Hamidrad et al., 2021). The objective of this study was to explore the trends and patterns of intervertebral disc and nucleus pulposus stresses under different lumbar curvature configurations using clinically feasible methods. The stress distribution and trend changes in lumbar intervertebral discs under different standing lumbar curvature configurations observed in this study are consistent with previous findings. The neutral posture is most beneficial for L4-L5 and L5-S1. After applying physiological loads representing the upper body weight to the FEMs, the peak disc stress shifted from the dorsal to the ventral side of the annulus fibrosus during forward flexion of the upper body compared to standing (Zander et al., 2003). The central region (nucleus pulposus) of the patient-specific, kinematically optimized L3-L5 finite element model exhibited the lowest stress (Du et al., 2024).

The transition from standing to sitting leads to a reduction in LL, primarily occurring at L4-S1, and an increase in intervertebral disc load (Hey et al., 2017; Patwardhan et al., 2021). It also causes a decrease in lumbar disc height (Zanola et al., 2024), The magnitude of LL is correlated with changes in the apparent diffusion coefficient of the anterior annulus fibrosus, suggesting an influence of compressive strain on fluid transport properties (Compagnon et al., 2022). The prevalence of lumbar disc herniation in Roussouly type 2 spines is three times that of healthy controls, indicating that flat back (reduced LL) and a lower pelvic incidence may play a potential role in the development of lumbar disc herniation (Ould-Slimane et al., 2025). When the LL varies within the range of 28°–66°, significant changes occur in disc compressive forces, shear forces, and facet joint loading (Müller et al., 2021). Variations in sagittal plane morphology among healthy individuals can introduce systematic bias in spinal load estimation (Borrelli et al., 2026). In this study, the higher peakvon Mises stresses and nucleus pulposus stresses in L4-S1discs in the relaxed sitting with a forward inclination, in a neutral position and with a backward inclination position among the five sitting postures are related to reduced LL. The long-standing uncertainty in the explanatory paradigm for disc degeneration has reinforced the hypothesis that seated lumbar disc loading may be detrimental to disc health. It is commonly accepted in academia that lumbar disc loads are higher during sitting than standing. Nachemson (Nachemson, 1966) suggested that sitting increases IDP by approximately 40% compared to standing. However, there is no direct evidence linking sitting posture to the etiology of disc degeneration, mechanical failure, or pain (Nachemson, 1975). Results from indirect analyses of spinal loading have questioned this. One study, using precise height measurement to qualitatively assess changes in spinal load via disc height reduction or increase, concluded that spinal load during sitting is lower than during standing (Althoff et al., 1992), similar findings were reported using another indirect method (Rohlmann et al., 1995). Another study (Wilke et al., 1999) implanted pressure sensors into the nucleus pulposus of the L4-L5 disc in volunteers, measuring an IDP of 0.5 MPa in relaxed standing, 1.1 MPa in standing forward flexion, 0.46 MPa in relaxed sitting without lumbar support, 0.55 MPa in upright sitting, and 0.83 MPa in sitting maximum forward flexion. The authors cautiously suggested that seated IDP might be lower than during upright standing. Experiments suggest that defects in the annulus fibrosus may be the initiation point for disc herniation (Wade et al., 2022), and disc degeneration shifts load from the nucleus pulposus to the annulus fibrosus (Wang X. et al., 2024). In advanced degeneration, the intervertebral disc no longer effectively transmits compressive loads, resulting in stress transfer to the annulus fibrosus and posterior elements, which reflects the progressive loss of the biomechanical function of the nucleus pulposus (Ardatov et al., 2026). The results of the present study also indicate that the peak von Mises stresses of the lumbar discs and nuclei pulposi during sitting are lower than during standing.

Sitting posture is associated with occupational low back pain. The von Mises stress in the disc under a flexed sitting posture is significantly higher than in other sitting postures, while an extended sitting posture leads to pronounced von Mises stress concentration in the posterior region of the inter-lamellar matrix (Zheng et al., 2023). Maintaining LL requires substantial trunk muscle activity. Without external support, LL can only be maintained for a relatively short duration before tending to disappear completely (Claus et al., 2009). Consequently, maintaining lordosis may lead to greater energy expenditure and increased fatigue (Nachemson and Morris, 1964). From this perspective, maintaining an upright sitting posture may be difficult. Relaxed sitting with lumbar support helps increase spinal height and enhances hydration of the lumbar discs (Naresh-Babu et al., 2024). Sustained trunk extension is beneficial for increasing spinal height and improving low back pain (Harrison et al., 2024). In individuals without low back pain symptoms or other spinal disorders, this can be considered a strategy for maintaining lumbar disc health. Seat backrests also help increase LL and reduce IDP (Andersson et al., 1979). Reclining against a backrest with co-contraction of trunk muscles can produce proper lumbar curvature, effectively stabilizing the lumbopelvic region and reducing local stress on passive structures (Watanabe et al., 2008). Lumbar support was simulated primarily by adjusting the LL to represent its corrective effect on spinal curvature, thereby indirectly reflecting the mechanical benefits conferred by the support (Gao et al., 2023). Future studies may further incorporate more refined contact mechanics models to directly simulate the reaction forces exerted by the lumbar support on the lower back, thereby improving simulation accuracy (Gao et al., 2023). Lumbar support sitting posture that maintains lumbar lordosis is associated with lower intervertebral disc stress. However, the causal relationship with reduced intervertebral disc load still requires further verification after refining the mechanical parameters of the support. A sitting posture that maintains lumbar lordosis may help reduce intervertebral disc stress; as for the effect of lumbar support, it needs to be comprehensively evaluated in conjunction with the actual contact between the support and the trunk and the load-sharing conditions.

Previous studies have utilized two-dimensional sagittal plane X-ray images to create simplified lumbar FEM (Kim et al., 2022). Building upon traditional FEM modeling based on CT data, this study recalibrated the model by rearranging the spatial positions of the lumbosacral vertebral bodies in the sagittal plane according to X-ray images taken in different postures. This approach aimed to simulate the gravitational collapse effect in commonly adopted standing and sitting postures in daily life. The resulting model’s spinal spatial structure and physiological curvature more closely approximate reality, overcoming the limitation that CT scans typically only capture supine position data, thereby improving modeling efficiency. In addition, the von Mises stress of the nucleus pulposus was analyzed. The modeling approach employed in this study is similar to the method reported by Hajnal et al. (2022), in which LL from standing radiographs was registered to a three-dimensional model based on supine CT images. Previous finite element studies have compared intervertebral disc stress and IDP under different sitting postures and have reached similar conclusions, namely, that an upright sitting posture or increased LL is associated with reduced loading of the lower lumbar spine (Cho and Han, 2023; Zheng et al., 2023). However, the study by Zheng et al. (2023) established only a porous finite element model of the L4-L5 motion segment, which may not be able to reproduce the true curvature of the L4-L5 motion segment under different sitting postures. The study by Cho et al. (2023) similarly used radiographic images of different sitting postures to model the vertebrae for each posture; however, this modeling approach was completed using specialized software, which limits its general applicability.

Several limitations in the study design should be acknowledged. First, and most crucially, this study modeled and tested data from only a single volunteer. The material parameters used in the FEM were validated in previous finite element studies rather than being derived from the specific individual. Therefore, the reliability and validity of the conclusions require cautious interpretation and validation with larger samples. Consequently, the significance of the data in this study is limited to observing trends of change. Second, indicators indirectly reflecting disc load, such as disc height, cross-sectional area, volume and disc deformation, were not analyzed. The predicted outcomes for different postures are based on common ranges of motion and cannot be generalized to extreme ranges. Moreover, the models did not consider muscle factors or changes in the mass and center of mass of body segments due to postural changes, as changes in the center of gravity and both global and local trunk muscle forces in flexed postures influence disc shear and compressive forces. The uniform load used in this study represents a necessary simplification and may underestimate the additional bending moments generated by shifts in the center of gravity under certain postures, particularly during forward flexion or twisting movements (Holsgrove and Ebisch, 2025). Third, this study employed a novel method to establish finite element models for different standing and sitting postures. The model’s spatial structure was primarily determined in the sagittal plane. Owing to the lack of three-dimensional imaging data, this study could not fully exclude the influence of axial rotation and coronal translation on disc stress and nucleus pulposus stress. Future studies should incorporate three-dimensional imaging techniques to improve the geometric fidelity of the model. Last but not the least, although the ROM validation demonstrated that the overall kinematic behavior of the model is reasonable, this does not guarantee the accuracy of the internal mechanical parameters of the intervertebral disc (Foltz et al., 2025). Future studies should, where possible, incorporate *in vitro* experimental or *in vivo* measurement data to directly validate disc stress and IDP, thereby enhancing the credibility of the model (Wang M. C. et al., 2024).

## 5 Conclusion

Lumbar curvature configurations significantly influences both the distribution and magnitude of von Mises stress in the L4-L5 and L5-S1 intervertebral discs and nucleus pulposus. Maintaining proper spinal alignment and avoiding postures that reduce lumbar lordosis are generally more favorable for lowering stress levels at these two segments. Specifically, in the standing position, the neutral posture is most beneficial for L4-L5 and L5-S1. In the sitting position, upright sitting in the neutral posture and relaxed sitting in the neutral posture with lumbar support are most favorable for L4-L5 and L5-S1; under these two sitting postures, the peak von Mises stress and nucleus pulposus stress at L4-L5 and L5-S1 remain at their lowest levels.

## Data Availability

The original contributions presented in the study are included in the article/Supplementary Material, further inquiries can be directed to the corresponding author.
