# Supplementary material for: Comparison of intervertebral disc and nucleus pulposus stresses under different lumbar curvature configurations
Source: Front Bioeng Biotechnol. 2026 Oct 23;14:1969400. doi: 10.3389/fbioe.2026.1969400 (PMC13739803; doi:10.3389/fbioe.2026.1969400)
Supplement: Supplementary file 1 [file Supplementaryfile1.docx]

Supplementary Material

# 1 Supplementary Figures

**S 1** Finite element models of standing with a forward inclination position (A), standing in a neutral position (B), standing with a backward inclination position (C).

**S 2** Finite element models of relaxed sitting with a forward inclination position (A), upright sitting in a neutral position (B), relaxed sitting with a backward inclination position (C), relaxed sitting in a neutral position (D), relaxed sitting in a neutral position with lumbar support (E).

**S 3** The Mises stress distribution of the nuclei pulposi under different standing lumbar curvature configurations. MAX: maximum.

**S 4** The Mises stress distribution of the nuclei pulposi under different sitting lumbar curvature configurations. MAX: maximum.
